# Deep learning based automated left atrial segmentation and flow quantification of real time phase contrast MRI in patients with atrial fibrillation

**DOI:** 10.1007/s10554-025-03407-9

**Published:** 2025-04-29

**Authors:** Justin Baraboo, Amanda DiCarlo, Haben Berhane, Daming Shen, Rod Passman, Daniel C. Lee, Patrick M. McCarthy, Rishi Arora, Dan Kim, Michael Markl

**Affiliations:** 1 Northwestern Biomedical Engineering, Chicago, Illinois USA; 2 Northwestern Radiology, Chicago, Illinois USA; 3 Northwestern Medicine, Cardiology, Chicago, Illinois USA; 4 Northwestern Medicine, Surgery, Chicago, Illinois USA; 5 737 N. Michigan Avenue Suite 1600, Chicago, Illinois 312694776 USA

**Keywords:** Atrial fibrillation, Real time phase contrast, Cardiac magnetic resonance imaging, Deep learning, Image processing

## Abstract

Real time 2D phase contrast (RTPC) MRI is useful for flow quantification in atrial fibrillation (AF) patients, but data analysis requires time-consuming anatomical contouring for many cardiac time frames. Our goal was to develop a convolutional neural network (CNN) for fully automated left atrial (LA) flow quantification. Forty-four AF patients underwent cardiac MRI including LA RTPC, collecting a median of 358 timeframes per scan. 15,307 semi-manual derived RTPC LA contours comprised ground truth for CNN training, validation, and testing. CNN vs. human performance was assessed using Dice scores (DSC), Hausdorff distance (HD), and flow measures (stasis, velocities, flow). LA contour DSC across all patients were similar to human inter-observer DSC (0.90 vs. 0.93) and a median 4.6 mm [3.5–5.9 mm] HD. There was no impact of heart rate variability on contouring quality (low vs. high variability DSC: 0.92 ± 0.05 vs. 0.91 ± 0.03, *p* = 0.95). CNN based LA flow quantification showed good to excellent agreement with semi-manual analysis (*r* > 0.90) and small bias in Bland-Altman analysis for mean velocity (-0.10 cm/s), stasis (1%), and net flow (-2.4 ml/s). This study demonstrated the feasibility of CNN based LA flow analysis with good agreements in LA contours and flow measures and resilience to heartbeat variability in AF.

## Introduction

Two-dimensional cine phase contrast (2DPC) magnetic resonance imaging (MRI) enables quantification of blood flow velocities for many applications [1, 2] including the assessment of hemodynamic parameters over the cardiac cycle, peak velocities, or cardiac output. Clinical applications include the assessment of flow and velocities in valvular regurgitation, [3, 4] valvular or vascular stenosis, [5 cerebrovascular diseases, [6, 7] or congenital heart disease [5, 8]. Studies have shown that flow and velocity quantification can also provide important diagnostic information in patients with atrial fibrillation (AF). For example, trans esophageal echocardiography studies have shown that decreased left atrial (LA) peak velocities or blood stasis are associated with greater risk of thrombus formation, demonstrating that altered LA flow conditions may impact downstream stroke risk [9, 10]. However, the application of standard 2DPC MRI techniques is challenging in this population. 2DPC MRI data is acquired during breath holding over several cardiac cycles and, therefore, cannot resolve beat-to-beat variations in LA blood flow velocities. Arrhythmia rejection techniques can be used to allow for cardiac gating in 2DPC acquisitions. However, they do so by outright rejecting arrhythmic heartbeats, which causes inefficient data acquisition, lengthened breath holds periods, and beat-to-beat variations in LA blood flow velocities remain unresolved.

To address these limitations, two-dimensional real-time phase contrast (2D RTPC) techniques have been introduced, which do not require cardiac or respiratory gating. To achieve real time flow imaging, advanced k-space acquisitions (echo-planar [11, 12], radial, [13, 14] and spiral [12, 15], parallel imaging (sensitivity encoding (SENSE) or generalized autocalibrating partially parallel acquisitions (GRAPPA)) and subsampling with advanced reconstruction methods (compressed sensing [16]) have been utilized for image acceleration and/or reducing motion artifacts. Subsequent flow quantification requires often manual, cumbersome, and time-consuming delineation of cardiovascular contours, limiting reproducibility and clinical translation. As an example, for a temporal update rate of 40ms and constant heart rate of 60 bpm (RR-interval duration = 1000ms), a user would have to manually delineate contours in 250 images (timeframes) for 2D RTPC data acquired in 10 heartbeats.

While there have been advances for semi-automated tools to perform vessel lumen contouring, these tools still require visual inspection and manual correction of contours and are packaged within proprietary software [17, 18]. Recently, artificial intelligence (AI) and deep learning tools have been employed to automate segmentation tasks in MRI processing. Convolutional neural networks have formed the building blocks for cardiac segmentation, including AI derived 2D ventricular contours in balanced steady-state free precession (BSSFP) short and long axis images [19–21]. The goal of this study was to build on these promising findings and develop an AI based workflow for fully automated 2D RTPC LA image contorting and flow analysis in patients with AF. We hypothesized that our AI based automated workflow would perform at the same level as human observers for LA contouring and hemodynamic quantifications. We also investigated the impact of heart rate variability on deep learning contour quality to assess the robustness of automated AI processing in the AF patient population.

## Methods

### Study cohort

This study was performed according to an institutional review board (IRB) approved protocol. Forty-four atrial fibrillation (AF) patients (Table 1) were retrospectively recruited and underwent cardiac MRI, including LA 2D RTPC with through-plane velocity encoding. Inclusion criteria was a history of AF or AF patients who had previously underwent an ablation ( > = 3 months prior to the study). No patients received an ablation during the study. Five AF patients underwent a second cardiac MRI as part of a test and retest reproducibility (mean time between scans 13 ± 5 days), for a total number of 49 AF patient data sets available for LA flow analysis. No patients had prior left atrial appendage closures, clipping, or ligation. Patients were excluded if they had MRI contraindications, were pregnant, had severe claustrophobia, were non-English speakers, or were unwilling or unable to give informed consent. All patients gave informed consent in writing.

**Table 1:**
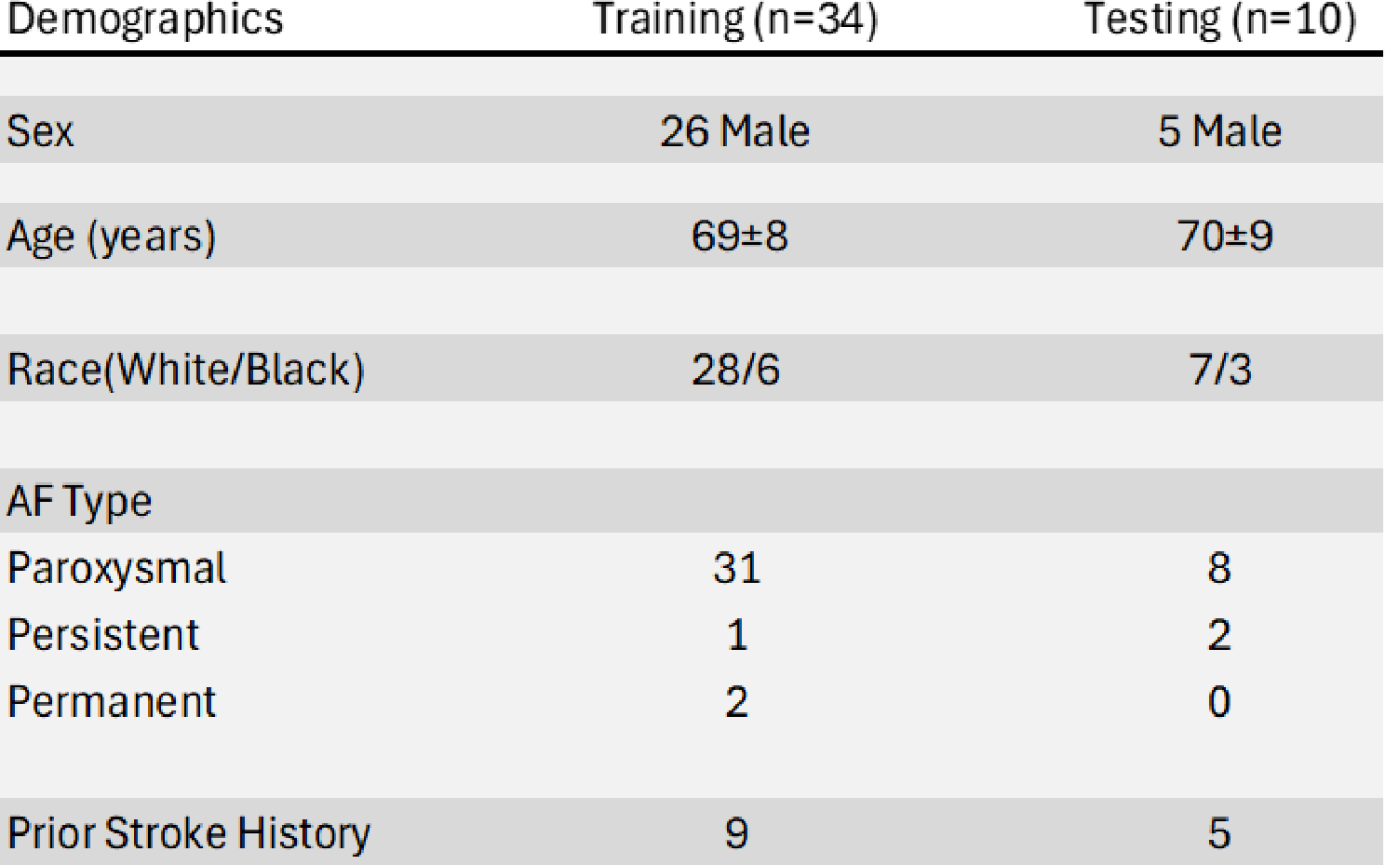
Patient demographics across training and testing. Race was self-reported by the patient

### MR imaging

**Fig. 1:**
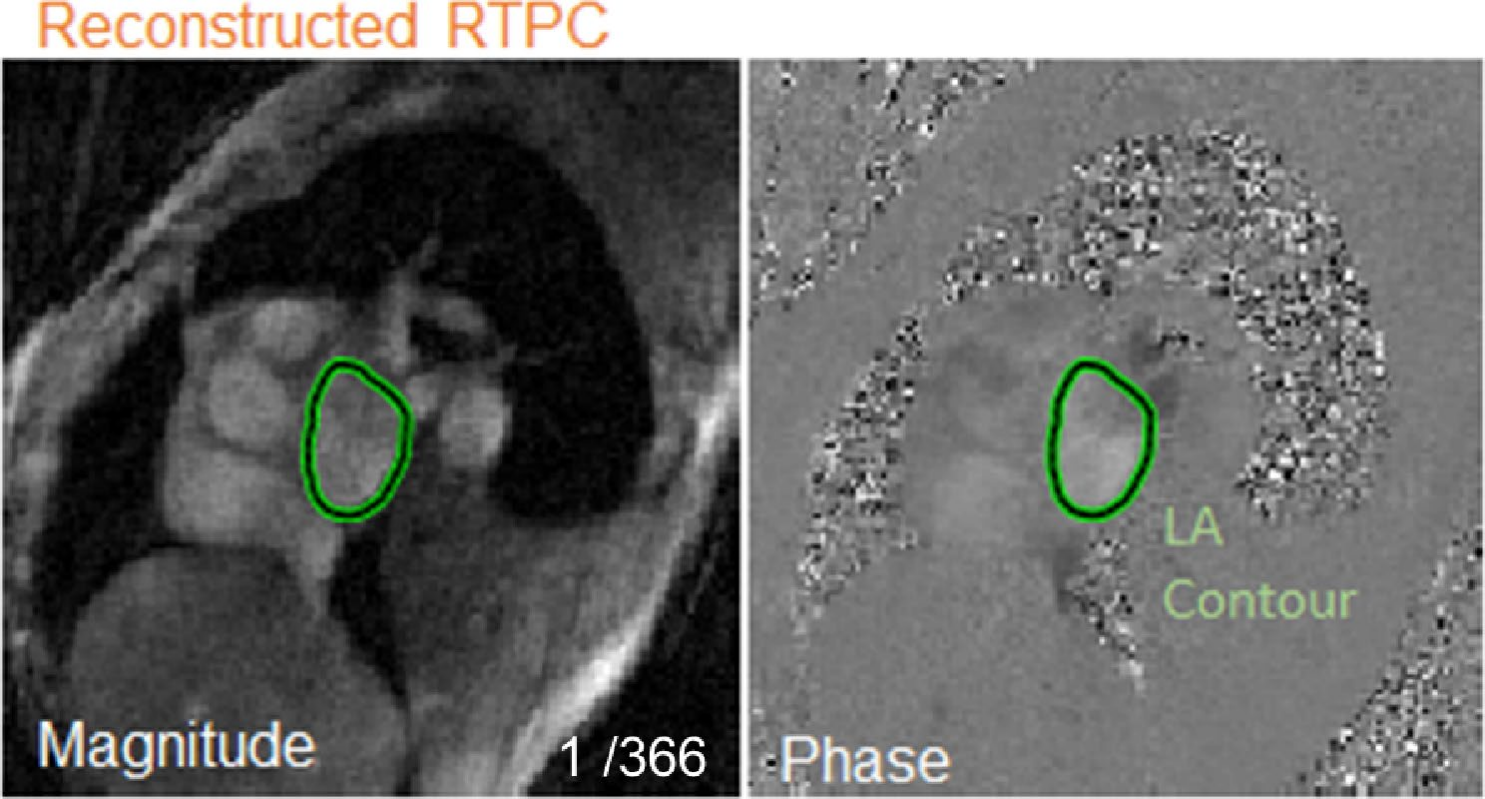
LA RTPC plane is placed parallel to the mitral valve. 2D RTPC raw data is reconstructed into magnitude and phase cardiac data based on acquisition time (366 frames total for this patient). The left atrium is contoured to quantify blood flow velocities. LA- left atrium

All subjects were scanned on a 1.5T MRI system (Siemens MAGNETOM Aera or Siemens MAGNETOM Avanto Erlangen, Germany). Cardiac MRI included anatomic and flow imaging of the LA. 2D RTPC imaging planes were placed at the mid LA oriented parallel to the mitral valve with through plane velocity encoding (Fig. 1). EKG R-waves detected by the scanner’s physiological measurement unit (PMU) were recorded in the 2D RTPC raw data header to delineate each heartbeat in subsequent analysis.

The 2D RTPC data were acquired using a previously described highly undersampled pulse sequence [14]. Data were acquired during free breathing using a gradient-echo sequence employing radial k-space sampling with golden angles (111.2461°) with the following acquisition parameters: venc = 60–70 cm/s, acquisition matrix size 192 × 192, field of view (FOV) = 288 mm x 288 mm, flip angle = 8°, spatial resolution = 2.1 mm x 2.1 mm, slice thickness = 7–8 mm, TE = 2.88 ms, TR = 4.25 ms, acceleration factor *R* = 28.0, and total acquisition time varied from 5 to 20 s.

As described previously in Haji-Valizadeh et al., undersampled k-space data were reconstructed offline using the GRAPPA operator gridding Golden-angle Radial Sparse Parallel (GROG-GRASP) method [14, 22]. Temporal total variation was used as the sparsifying transform with normalized regularization weight 0.0078 (relative to the maximum signal of the time-averaged image) and 22 iterations. Each image was reconstructed with 5 radial k-space lines per time frame, with interleaved reference and velocity-encoded acquisitions for each k-space line, resulting in an effective temporal resolution of 42.5 ms (= TR x 2 × 5).

### Semi-automated RTPC flow analysis

For LA flow quantification, a semi-manual processing pipeline was used to delineate the outline of the LA on 2D RTPC magnitude images for all time frames for each patient (Fig. 1). The LA was manually contoured on the first time frame to determine a seed region as input for a magnitude intensity-based region growing algorithm (Matlab, Mathworks). This seed region was then propagated to all time frames and region growing was subsequently applied. Next, each time frame was manually reviewed, and a human observer could modify the LA contour, typically removing excess contoured areas encroaching into the pulmonary vein, left ventricle, or aorta. Alternatively, the observer could add to the contoured area, typically near the LA wall. These LA contours served as ground truth labels for training and testing of the deep learning network for fully automated LA contouring. Furthermore, to assess inter-observer variability, the semi-automated analysis was repeated for twelve patients by a second observer, blinded to the results of the first reader.

### Convolutional neural network design for 2D RTPC LA contour delineation

**Fig. 2:**
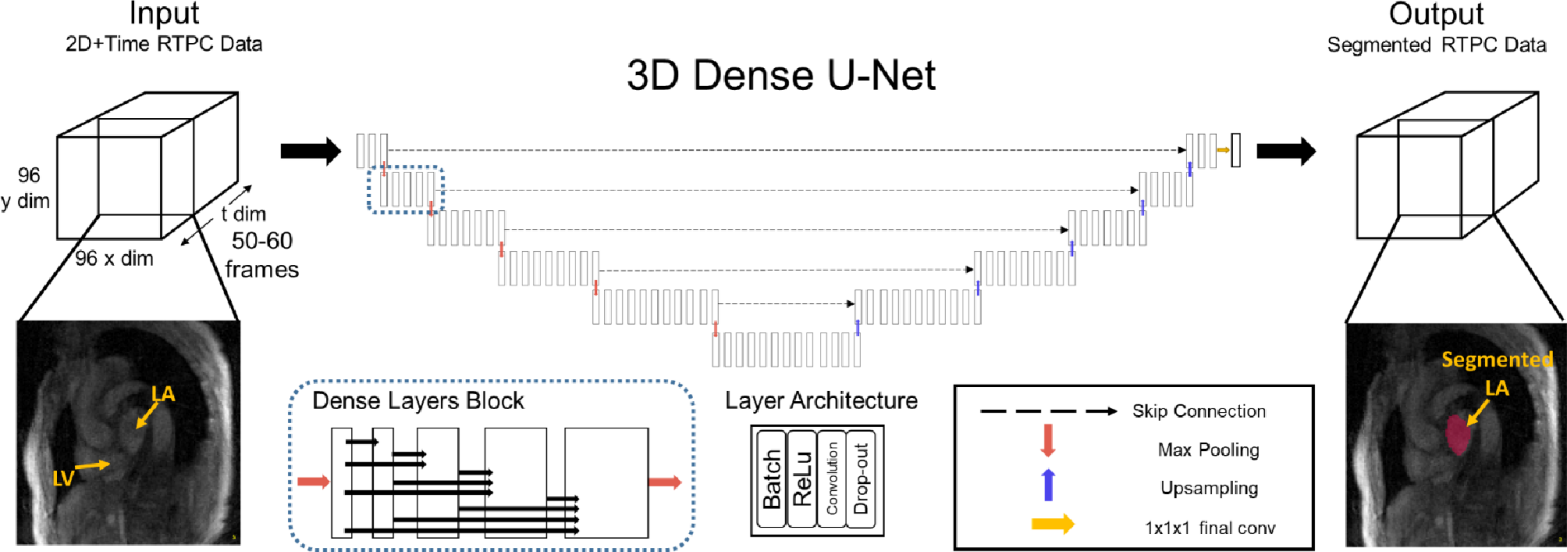
RTPC magnitude volume data (2D image data + time) is preprocessed and inputted into a dense U-net architecture convolutional neural network. The network features 2,4,6,8, 12 dense (outputs of each layer feed forward as inputs to subsequent) layers per layer with each layer consisting of batch, relu, convolution and drop out with constant feature map size of 12. Max-pooling was utilized for down sampling within the encoder structure and transposed convolution within the decoder structure. The final layer consisted of a 1 × 1 × 1 convolution and softmax function to generate a probability of each voxel (2D spatial + time) of the magnitude input image volume belonging to the left atrium or the background for each time frame

A 3D U-Net network [23] with DenseNet-based dense blocks [24], replacing the typical U-Net convolutional layers was utilized to segment the LA (Fig. 2). This network was adapted based on a previous network for 3D segmentation of the thoracic aorta [25]. Previously validated hyper parameters for the network (network depth, layer design, optimizer, and learning rate) were used, saving the computational effort of performing a nested cross validation scheme. Underscoring, the model weights, themselves, were zeroed and retrained. The hyper parameters used: a constant learning rate 0.001, ADAM optimizer, max pooling, a dropout rate of 0.1, skip connections from decoder to encoder sections, and channel size of 12. Dense blocks concatenated all prior feature maps for use as inputs to subsequent layers, which allowed for efficient feature reuse. Max-pooling was utilized for down sampling within the encoder structure and transposed convolution within the decoder structure. The final layer consisted of a 1 × 1 × 1 convolution and softmax function to generate a probability of each voxel (2D spatial + time) of the magnitude input image volume belonging to the LA or the background, and the LA contour was generated by selecting the class with the highest probability. A composite loss function composed of minimizing the equal sum of the softmax-cross entropy and complement of Dice score during training was utilized. During training and validation, an identical network with long short term memory (LSTM) blocks was trained and compared to the above network. Deep learning networks were developed in *Python 3.6.8* (Python Software Foundation, Beaverton, OR) using *Tensorflow* 1.12.0 (Google, Mountain View, CA). Training time was 36 min. Training and testing of the deep learning network was performed on a NVIDIA GeForce GTX 1080 with an Intel i7-8700k processor.

Reconstructed 2D RTPC data were automatically pre-processed prior to network input. Each patient’s 2D RTPC time series of magnitude images can be denoted as $\:P({I}_{1}\dots\:{I}_{t}\dots\:{I}_{T})$ where $\:{I}_{t}$ represents a magnitude image at timepoint *t* where $\:t\in\:[1\dots\:T]$ and T is the total number of time frames for a patient *P*. Every magnitude image per patient was automatically center cropped to a fixed dimension of 96 × 96. Next, the data was automatically broken into multiple 50 time frame blocks with a 20 time frame overlap between neighboring blocks given by:

$$\:{b}_{j}\left(I\right)=\left\{P\left({I}_{i}\right)\right|\:i\in\:\left[30\left(j-1\right)+1\:.\:\:\:.\:\:\:.\:\:30\left(j-1\right)+51\right]\}$$

Where b is the j^th^ block of 50 time frames. If the final block was less than 50 time frames, it was concatenated to the previous block. Frame overlap between blocks served to increase the number of training data set inputs and reduce input size compared to full patient data input, reducing GPU requirements. 50 time frames of data corresponded to a total time of 2125ms, covering multiple heartbeats. Images were normalized and rotations (flips and mirroring) were used for data augmentation.

### Convolutional neural network training and testing

Ground truth 2D LA contours were generated in 49 RTPC scans with a median of 355 RTPC images (time frames) per scan [q1 q3 quartiles: 173–366]. A total number of 15,307 cardiac 2D RTPC images with semi-manual derived LA contours were available as ground truth for CNN training, validation, and testing. Five-fold cross validation schema was utilized at the patient level (patients with rescans had both scans assigned to training or validation). After validation, the network was retrained with all available data before testing. The final test consisted of 10 patients (held out from all training and validation).

### Convolutional neural network performance

Dice score (DSC) and Haursdorff Distance (HD) were used to assess LA contouring accuracy per time frame, defined as:

$$\:DSC\left(X,Y\right)=\frac{2|X\cap\:Y|}{\left|X\right|+\left|Y\right|}\:,\:\:HD\left(X,Y\right)=\underset{\text{x}\in\:\text{X}}{\text{max}}\left\{\underset{y\in\:Y}{\text{min}}\left(d\right(x,y\left)\right)\right\}$$

where X and Y are the binary semi-automated and deep learning contours for a time frame. d(x, y) is distance between points x and y for x in X and y in Y. DSC was also used to quantify differences between human observer LA contouring for analysis of inter-observer variability. DSC and HD were aggregated over all time frames, tested for normality, and reported as a mean with standard deviation (or median with q1 and q3 quartiles if determined non-normally distributed). Over-segmented voxels (identified as LA by the model but not the human observer) and under-segmented voxels (identified as background by the model but not the human observer) were found. The relative over and under-segmented area to the LA contour was quantified for each frame and each patient. Signal to noise ratio (SNR) was calculated for each patient in the testing set to measure image quality. Correlation between DSC and SNR was calculated to test performance of the network against image quality. To determine if performance was impacted by which part of the cardiac cycle the network performed in, mean DSC between systolic frames and diastolic frames for each patient for each heartbeat in the testing set.

### Left atrial flow quantification

Both semi-automated and convolutional neural network LA contours were used to quantify LA peak and net flow, LA peak and mean velocities, and LA blood stasis. For each AF patient, a series of cardiac cycles were identified by consecutive RR signals recorded by the MRI scanner’s physiological monitoring unit EKG signal during 2D RTPC data acquisition. For each cardiac cycle, mean LA velocity was calculated as the average over all LA velocities per cardiac cycle. Peak velocity was calculated as the mean of the top 5% of all velocity values to eliminate spurious noisy pixels. Flow-time curves (blood flow in the direction of the plane) were derived for each patient and peak and net flow were calculated for each cardiac cycle. Blood stasis was calculated based on previously reported strategies as the percentage of cardiac time frames per cardiac cycle below a threshold selected to be 10 cm/s for three-directional velocity encoding [26]. For the present study the threshold was adjusted to 5.8 cm/s (=$\:\frac{10}{\surd\:\left(3\right)}$) to account for single-directional vs. three-directional velocity encoding.

### Heart rate variability analysis

To assess the impact of heart rate variability in AF on LA contouring accuracy, a grouped analysis of lower vs. high heart rate variability AF patients was performed. Heart rhythm variability was quantified by calculating the coefficient of variation (RR standard deviation / RR mean length) based on the PMU EKG measured RR-interval durations during the 2D RTPC acquisition. Based on previously reported findings, a coefficient of variation greater than 0.24 was employed as a threshold for low vs. high heart rate variability. [27] Both the final test set and aggregate per k-fold validation set results were analyzed. The heart rates for the final test set were calculated and correlated against performance.

### Statistical analysis

LA mean velocity, peak velocity, net flow, and stasis were compared between semi-automated and deep learning generated LA contouring. A Lilliefors test was used to determine the normality of each distribution and they are reported as mean ± std if normal or median and q1 and q3 quartiles if not. Differences were assessed with a paired t-test if deep learning and semi-automated distributions were normal or a Wilcoxon signed rank test if not, at the α = 0.05 level. Pearson correlation was used to quantify the linear relationships of each flow measure between the semi-automated and automated processing pipelines. Bland-Altman analysis was used to quantify the bias between techniques and their limits of agreement (LoA). Differences in DSC between high heart rate and low heart rate variability were assessed using a t-test at the α = 0.05 level.

### Data and code availability

The trained network model and execution code is available publicly (https://github.com/JustinBaraboo/2DRTPC_Segmentation_LA). The imaging data will not be made publicly available.

## Results

### 2D RTPC analysis time

Testing set LA data analysis included 10 2D RTPC data sets covering a total of 188 heartbeats and 3,571 cardiac 2D RTPC time frames. Analysis time for semi-automated LA contouring with manual corrections was for observer 1: 72 ± 23 min per patient (timed for seven 2D RTPC data sets with a time series of 366 images each) and for observer 2: 71 ± 21 min (timed for five 2D RTPC data sets with 366 images each). It took 5 s for fully automated deep learning LA contouring (timed for all 49 2DRT data sets).

### Convolutional neural network performance – LA contours

The five-fold cross validation strategy resulted in 26–29 unique patients in each training fold and 5–8 unique patients for each validation fold. After the spatial cropping and temporal blocking preprocessing steps, each training fold had 263–311 blocks of input for training, prior to data augmentation, consisting of 13,507–16,247 2D RTPC images, and 36–88 blocks of testing cases, or 1868–4608 2D RTPC images, per fold. The 10-patient final testing set consisted of 3,571 frames.

**Fig. 3:**
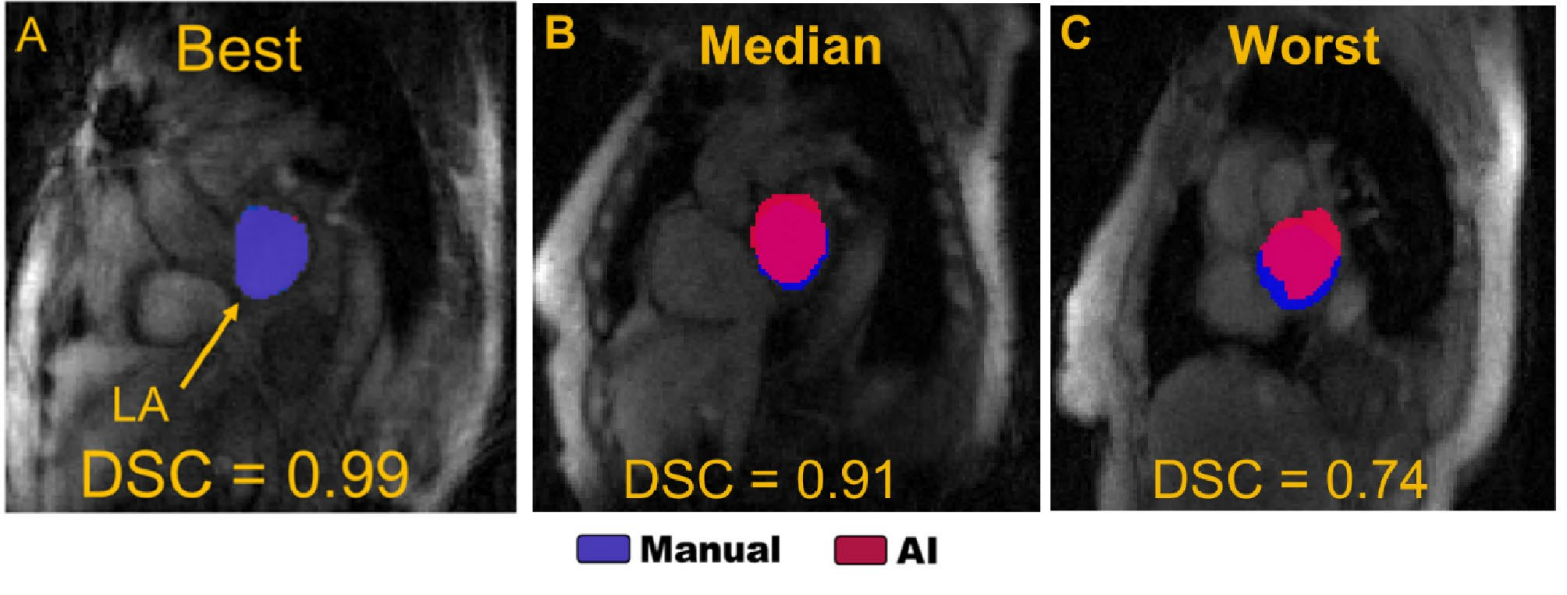
Best (**A**), median (**B**), and worst (**C**) dice score performing frames over the entire patient cohorot. Semi-manual (blue) is diplayed over AI (red) contours. Each frame comes from a different patient

Fully automated AI based LA contour delineation had an average per frame DSC of 0.90 [0.88–0.92] across the entire test set data (*n* = 3,571frames), indicating excellent agreement with the ground truth semi-automated analysis. Across subjects, the average standard deviation DSC between frames within the subject was 0.02 ± 0.01. Per frame HD was 4.6 mm [3.5–5.9 mm], approximately a distance of 2 voxels [1.7–2.8 voxels. LA contours agreed well between AI vs. semi-automated analysis throughout all time frames with the exception of 39 individually low DSC (< 0.80) time frames (1.1% of all 2D RTPC images). The best, median, and worst DSC score cardiac frames across all patients are shown in Fig. 3 where poorer DSC agreement was due to over contouring of the LA, including the aorta (Fig. 3c) or pulmonary artery. Inter-observer average DSC was 0.93 ± 0.04 (*n* = 2,909 frames), demonstrating similar performance for semi-automated human and deep learning-based analysis. We found that, on average, there was an 11 ± 7% over-segmentation and an 8 ± 6% under-segmentation. The mean SNR of the images for each patient in the testing set was 4.2 ± 0.9. There was no significant correlation between performance DSC and SNR (*p* = 0.45). We did not find a significant difference in systolic or diastolic DSC performance for any patient (lowest *p* = 0.13, range of p-values [0.13–0.98]).

### Impact of heart rate variability on neural network performance

In the final 10 test sets, 3 patients (3 scans) demonstrated higher HRV. Due to this low sample size, statistical testing of differences was not performed. We do report the values for the within patient mean DSC values for the HRV subgroup (0.86,0.88,0.92) and LRV test set group (0.90 ± 0.02). The test set heart rates ranged from 42 to 110 bpm (81 ± 20 bpm). There was no significant correlation between heart rate and DSC scores in the testing set (*p* = 0.38). In the aggregated k-folds validation sets, a total of 9 AF patients (11 2D-RTPC scans) demonstrated high heart rate variability, and 26 AF patients (28 2D-RTPC scans) were found to have low heart rate variability. DSC scores for AI vs. semi-automated analysis were similar for both low and high heart rate variability (0.92 ± 0.05 vs. 0.91 ± 0.03, *p* = 0.95).

### Convolutional neural network performance – LA flow quantification

**Fig. 4:**
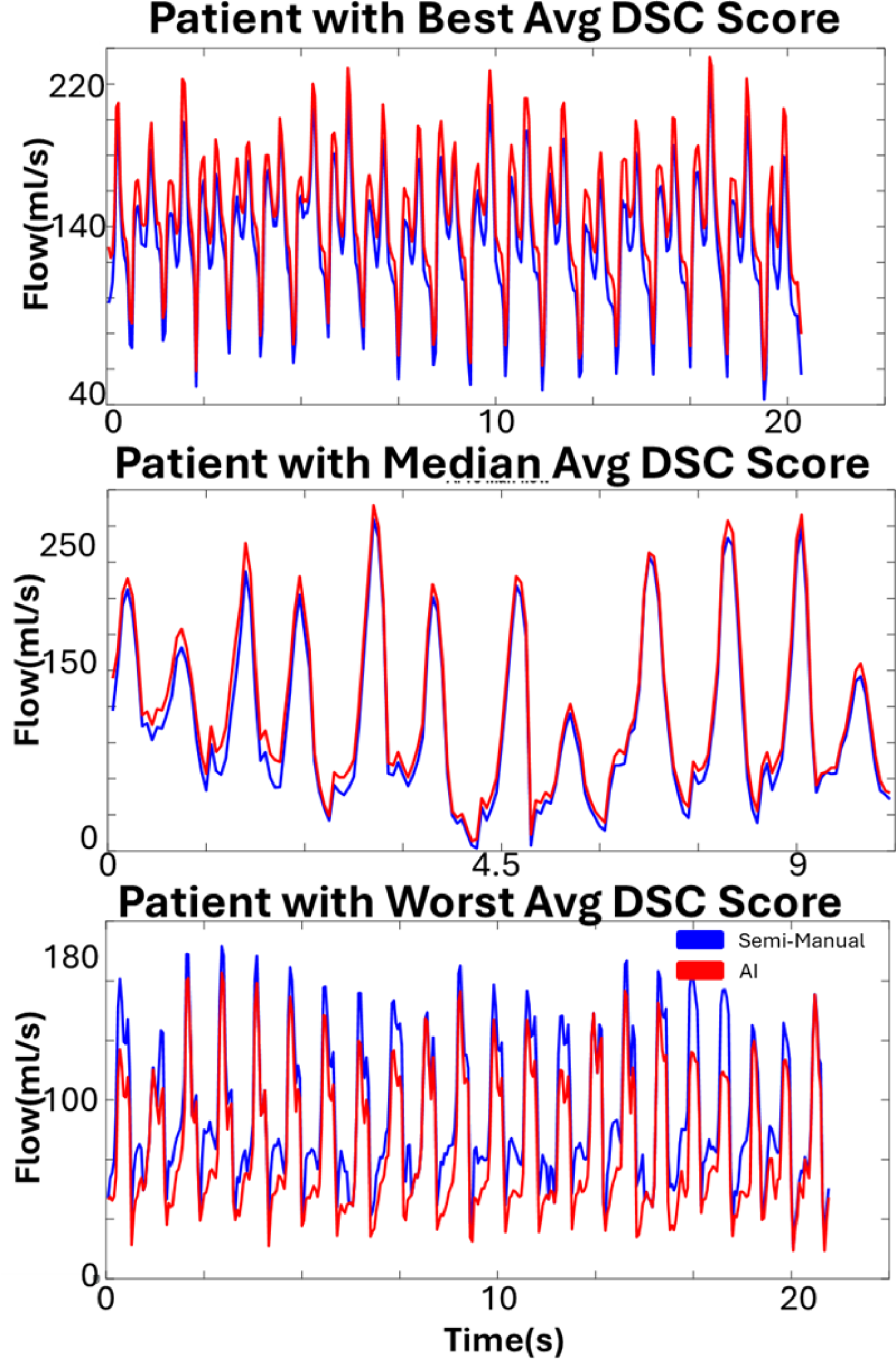
Flow curves for the patients having the best (top), median (middle), and worst (bottom) dice score frame. AI (red) displayed excellent agreement with Semi-Manual (blue) flow values over time, despite low performing frames

As summarized in Table 2, AI derived LA peak velocities, mean velocities, blood stasis, and net flow per cycle demonstrated excellent agreement with semi-automated analysis (*r* = 0.80, *r* = 0.94, and *r* = 0.94, *r* = 0.94, all *p* < 0.05). LA flow-time curves are shown for AF patients with the best, median, and worst DSC scores (Fig. 4).

**Table 2:**
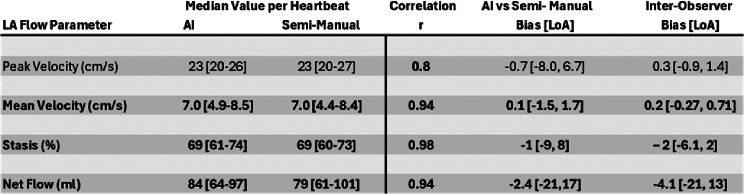
Left: median and interquartile ranges for AI derived and vs. semi-manual analysis of LA flow parameters in all 39 AF patients. Right: agreement between AI and manual was evaluated by pearson correlation and Bland Altman analysis. For comparison of AI vs. human performance, Bland Altman analysis of inter-observer variability is shown in the right most column. Bold indicates a significant difference between AI and semi-Manual analysis via Wilcoxon signed ranked testing at the α = 0.05 level

**Fig. 5:**
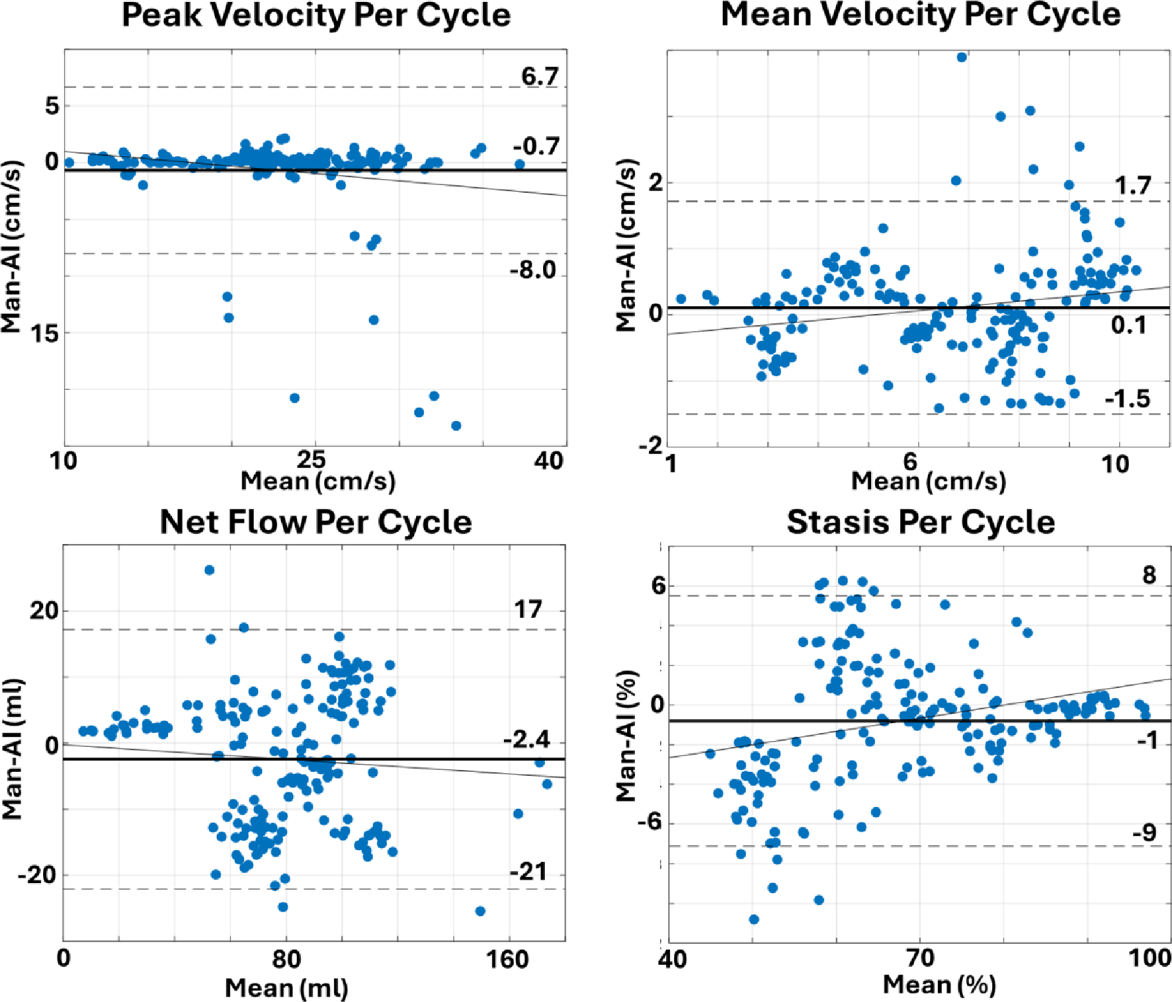
Bland Altman analysis of agreement between semi-manual and AI per heartbeat measurement of peak velocity, mean velocity, net flow, and stasis

LA peak velocities showed excellent agreement (bias = -0.7 cm/s, LoA = ± 7.3 cm/s, *p* = 0.15) between semi-manual and AI workflow (Fig. 5, and Table 2). There were 14 heartbeats with > 4 cm/s overestimated peak velocities in the AI analysis due to the AI inappropriately contouring a surrounding high velocity vessel.

Bland Altman analysis demonstrated a small but significant underestimation of LA mean velocity by AI (bias = -0.10 cm/s, *p* < 0.01, LoA of ± 1.6 cm/s, Fig. 5, and Table 2). Mean absolute stasis difference between the two techniques was 2.9% (bias = 0.1%, LoA of ± 8%, *p* < 0.01 Fig. 5, and Table 2). Mean absolute net flow per cardiac cycle differences between the techniques was 4.9 ml/cycle and had a small, but significant, overestimation by the AI (bias = -2.4 ml/cycle LoA of ± 19 ml/cycle).

As summarized in interobserver Bland Altman analysis revealed a small but significant bias between observers for peak velocities (bias = 0.26 cm/s, LoA of ± 1.1 cm/s), mean velocity (bias = 0.22 cm/s [2.9% of mean], LoA of ± 0.49 cm/s), stasis (bias = -2% [5% of mean], LoA of ± 4.1%), and net flow (bias = -4.1 ml/cycle [3.5% of mean], LoA of ± 17 ml/cycle).

## Discussion

The findings of our study demonstrate the feasibility of deep learning to accurately delineate LA contours in 2D RTPC imaging across multiple heartbeats. The deep learning results agreed well with semi-automated LA contour shapes, which were at human semi-automated interobserver level. Deep learning also displayed excellent agreement of hemodynamic indices with semi-automated analysis. While there were statistically significant bias differences between methods for net flow, stasis and mean velocity measures, these differences were small (< 2% of the mean in Bland Altman analysis) and less than the human inter-observer’s bias for net flow, stasis, and mean velocity. Deep learning vs. semi-manual limits of agreement were comparable to two human observers for stasis and net flow. However, there were larger variations in peak velocity and mean velocity limits of agreement between deep learning and the semi-manual ground truth. This may be due, in part, to their sensitivity of including higher velocity vessels. Previously reported aortic interobserver net flow quantifications were roughly 8–10 ml/cycle^28,29^ which are lower than what we measured in the atrium (either between interobservers or deep learning). This may be due to the more complex atrial geometry, particularly disagreement at the wall. There was no impact of heart rate variability on deep learning contour quality, underscoring the potential of this AI based automated processing for applications in the AF patient population, but a larger testing set including more HRV patients is necessary to confirm this.

Analysis of 2D RTPC imaging greatly benefits from automated processing due to the large number of time frames reconstructed. However, there has been limited applications of automated processing methods for this technique. Commercial software has been utilized for automated 2D RTPC segmentation of the aorta and superior vena cava [17, 18]. However, in these studies, the authors reported the need to manually adjust poorly contoured frames for as many as 27.4 ± 10.5% of images per patient. A previous semi-automated tool for 2D RTPC contouring of the aorta achieved DSC scores of 0.89 ± 0.04 compared to a manual observer [28]. These results compare similarly to our performance in the atrium (0.92 ± 0.04). For non-real time 2D phase contrast imaging, aortic segmentation at the valve achieved a mean DSC score of 0.94 using a residual U-Net with 2D magnitude image input [30]. Herment et al. used a 3D (2D images + time) deformable surface model to automate 2D phase contrast segmentation of the ascending and descending aorta, achieving a high mean DSC score of 0.95 [31]. They detail that accounting for time may have helped to ensure accurate segmentation, instead of frame by frame LA contouring which may be utilized by a 2D CNN network. By incorporating time, they found their segmentation to be more consistent with fluid and wall movement, albeit with a surface model algorithm. While not directly quantified in this study (against a 2D U-net implementation), our similar DSC results for the novel LA application may be due in part to inputting and learning from a 2D + time volume as opposed to 2D per frame segmentation. Overall, our model performed at the same level or better compared to these studies in terms of atrial shape segmentation.

Deep learning advances for the left atrium have been confined to 2D cine LA contouring or 3D gadolinium enhanced LA segmentations [32]. Previous studies have reported deep learning LA contouring on 2D cine long axis data covering the left atrium. Bai et al. performed comprehensive heart segmentation for ventricular (short and long axis) and atrial (long axis: 2 and 4 chamber views) for UK Biobank data (*n* = 4,875) using fully convolutional networks [19]. Their study achieved mean LA DSC Scores of 0.93 (2 chamber) and 0.95 (4 chamber). Zhang et al. utilized a deep convolutional layer network and unscented Kalman filtering to contour the left atrium in 2, 3, or 4 chamber views achieving mean DSC scores of 0.94, 0.94 and 0.90, respectively [33]. Our convolutional neural network had comparable DSC scores to these values with a mean score of 0.92 compared to a semi-automatic human analysis workflow. Achieving similar DSC score results is encouraging as BSSFP based cine acquisitions are known to provide high cardiac blood-tissue contrast, i.e. better-quality input data compared to our 2D RTPC data. Our network and processing differ from the previous examples through use of 3D volume input (2D images + time) instead of 2D image input.

Clinically, 2D RTPC may be underutilized in the setting of AF patients. While there has been reconstruction advances and validation studies post processing and analysis remains cumbersome [29, 34–36]. LA stasis and peak velocity quantifications, barring inclusion of the aorta or outflow tract, were the most robust parameters. These measures of slower blood flow may be important for understanding atrial myopathy or outcome risk in these patients [37, 38]. Further studies are necessary to determine which values and cut off may be important for understanding these conditions, but automated quantification is a necessary component for large scale studies.

### Limitations

Our study has several limitations. First, two of the AF patients in our study cohort had > 4 cm/s overestimated peak velocities in the AI analysis due to excess contouring including the aorta or left ventricular outflow tract. Indeed, the left atrium is surrounded by many different vessels and the left ventricle, where small contouring errors can lead to larger velocity measurement error. These errors were not apparent in the interobserver analysis, likely due training and observation while segmenting (learning to be able to distinguish the aorta and the outflow tract from the LA). We speculate that the observed differences in stasis between AI and semi-automated analysis were related to consistent under-contouring of the LA wall by one of the techniques. This was also evident for inter-observer analysis, where one reader consistently contoured less of the LA wall leading to reduced stasis. Future studies should investigate whether incorporating velocity information as a separate input channel in the convolutional neural network or within the loss function can improve AI performance and avoid the inclusion of neighboring vessel or compartment with different flow velocities.

Second, only one 2D RTPC plane placed in the left atrium parallel to the mitral valve was examined in this study. Future studies should assess the performance of our AI network architecture (either directly with the current network or retrained) for other atrial plane locations and orientations to enhance the generalizability of the AI.

Third, the deep learning network was validated and tested using data only from a single site and scanner vendor, which may limit generalization to other sites and scanners. Thus, for assessing model generalizability, a larger sample size with external validation is necessary. There was no analysis on the impact of changes in data acquisition parameters, such as spatial resolution, temporal resolution, field strength, or compressed sensing acceleration factors. We also did not tune certain hyperparameters such as cropping size (or no cropping at all). Further, other data augmentations that may simulate this were not utilized. We speculate that since we trained only on the magnitude data, that the network may translate to similar relative contrast 2D RTPC; however, we have only tested on this specific protocol and reconstruction. Finally, we did not train other machine learning or deep learning models for this data set to benchmark our deep learning performance. Future studies should thus systematically explore the performance of AI based LA flow analysis across different sites, MRI system vendors, and MRI acquisition protocols [39]. Adaptation of the network may benefit from an explanation of how the network is able to derive its contouring, but this was not investigated.

Finally, our database for training was small on the patient level. While we had many frames of data to train with, the variation seen within these patients may not account for the total variation within all atrial fibrillation patients and thus may limit the network. Further, frames within a patient are likely to be highly correlated which may affect model training, possibly leading to overfitting and effecting model translation. We also did not assess other cardiac or valvular diseases, where the model may not generalize. This study may be insufficient to determine the impact of heart rate variability on the model’s performance due to too few high heart variable patients in the testing set. Only one method of determining heart rate variability was utilized in this study where other subgrouping methods may yield different results.

## Conclusion

A deep learning tool was developed for automatic LA contouring and flow analysis for 2D RT PC MRI. The technique performed at human interobserver level for LA contouring and flow analysis, independent of heart rate variability in AF. AI based LA flow analysis shows promise for increasing data analysis efficacy and may allow for better clinical translation of 2D RT PC MRI applications.

## Data Availability

The trained network model and execution code is available publicly on github (https://github.com/JustinBaraboo/2DRTPC_Segmentation_LA). The imaging data will not be made publicly available.

## Abbreviations

2D: Two-dimensional
AI: Artificial intelligence
AF: Atrial fibrillation
CS: Compressed sensing
CNN: Convolutional neural network
DSC: Dice score
FOV: Field of view
GRAPPA: Generalized autocalibration partially parallel ascquisition
GROG-GRASP GRAPPA: Operator gridding golden angle radial sparse parallel
HD: Hausdorff distance
LA: Left atrium
LoA: Limits of agreement
LV: Left ventricle
PMU: Physiological monitoring unit
RTPC: Real time phase contrast
SENSE: Sensitivity encoding
TE: Echo time
TR: Repetition time
VENC: Velocity encoding
